# Absence of serological or molecular evidence of *Leptospira* infection in farmed swine in the Hong Kong Special Administrative Region

**DOI:** 10.1016/j.onehlt.2021.100321

**Published:** 2021-08-30

**Authors:** Kate J. Flay, Dan A. Yang, Michael T. Wilson, Song H. Lee, Vidya Bhardwaj, Fraser I. Hill, Dirk U. Pfeiffer

**Affiliations:** a Jockey Club College of Veterinary Medicine and Life Sciences, City University of Hong Kong, Kowloon, Hong Kong, China; b CityU Centre for Applied One Health Research and Policy Advice, City University of Hong Kong, Kowloon, Hong Kong, China; c CityU Veterinary Diagnostic Laboratory Co, Ltd, City University of Hong Kong, Kowloon, Hong Kong, China

**Keywords:** *Leptospira*, Swine, Hong Kong Special Administrative Region (HKSAR), Microscopic Agglutination Test (MAT), Serology, Polymerase chain reaction (PCR), HKSAR, Hong Kong Special Administrative Region, MAT, Microscopic Agglutination Test, FAO, Food and Agriculture Organisation of the United Nations, OIE, World Organisation for Animal Health, PCR, Polymerase Chain Reaction, PDF, probability density function, VDL, Veterinary Diagnostic Laboratory, WHO, World Health Organisation

## Abstract

Leptospirosis is an important zoonotic disease with several maintenance host species including swine. A cross sectional survey was undertaken between January to October 2020 to investigate the prevalence of leptospirosis in farmed swine in the Hong Kong Special Administrative Region (HKSAR) of China. Serum samples were collected from swine on seven farms (15 swine per farm; ten multiparous sows and five twelve-week-old weaners), while kidney samples were collected from 64 swine submitted for routine post-mortem (26 farms; average 2.4 swine per farm, range 1–6). Microscopic agglutination tests (MAT) to a panel of 24 *Leptospira* antigens did not reveal any evidence of seroconversion at a titre of 1:100. Polymerase chain reaction (PCR) testing of the kidney samples for *Leptospira* DNA did not detect any evidence of infection. Bayesian methods were used to compute the probability that the leptospirosis prevalence in farmed swine in the HKSAR was <3%, given none of the 105 swine sampled were positive on the MAT. The results of this study demonstrate no serological or molecular evidence of leptospirosis in farmed swine in the HKSAR. Subsequent statistical analysis supports the conclusion that the prevalence of leptospirosis in farmed swine in the HKSAR is negligible at present.

## Highlights

• First study to describe leptospirosis status of farmed swine in the HKSAR
• No serological or molecular evidence of leptospirosis in farmed swine in the HKSAR
• At this point in time, leptospirosis is not a priority for swine veterinarians and commercial farmers in the HKSAR.

## 1 Introduction

Leptospirosis is a disease caused by a variety of serogroups of the Gram-negative spirochete *Leptospira* [1]. Infection is derived primarily by direct or indirect contact with infected urine from maintenance hosts [2]. Zoonotic transmission of leptospires from infected animal hosts to humans can occur [[3], [4], [5]], contributing to morbidity and mortality among human populations [6].

Human leptospirosis is a health concern in many countries, particularly in tropical and subtropical areas where livestock are present [3,5]. In the Hong Kong Special Administrative Region (HKSAR) leptospirosis is a notifiable disease, with between 3 and 7 human cases reported per annum from 2015 to 2019 [7]. However, it has been suggested that human cases of leptospirosis in the HKSAR may be underdiagnosed due to the non-specific presenting signs and a possible lack of awareness of local healthcare professionals [8]. Internationally, farmed swine have been associated with zoonotic *Leptospira* infection [4,9], with swine producers and slaughterhouse workers at particular risk of infection [10].

Swine are maintenance hosts for a range of *Leptospira* serovars including Canicola, Grippotyphosa, Icterohaemorrhagiae, and Pomona [11] while swine disease is associated with serovars Australis, Canicola, Grippotyphosa, Hardjo, Icterohaemorrhagiae, Pomona and Tarassovi [12]. Swine leptospirosis can be an endemic infection with little evidence of disease, but when first introduced to a naïve herd, or a herd with waning immunity, infertility, abortion, neonatal death and weakness can ensue, while leptospires can persist in the kidney and genital tract of carrier swine [12].

*Leptospira* enter a susceptible host via damaged skin or through the conjunctival mucosa and circulate in the bloodstream for about 10 days [13]. Leptospires then localise in the proximal renal tubules, are voided in the urine and can persist for long periods [14]. Leptospires can be detected in the blood early in the course of infection, then in the kidneys or urine later in infection by culture or molecular methods [15]. After a serological response is mounted to infection (or vaccination), titres are detected by microscopic agglutination methods to the various infecting serogroups [16,17]. Leptospirosis infections can result in macroscopic white lesions in the kidneys of infected swine seen at slaughter, but this is an insensitive method of predicting infection in herds [18,19].

Seroconversion, widely considered as titres of ≥1:100 in the microscopic agglutination test (MAT), has been reported in populations of farmed swine in various countries. A recent serological survey from Germany [20] reported a seroprevalence of 20.2%, however it is important to note that this study used passive surveillance, testing 29,829 serum samples submitted to a diagnostic laboratory, with >85% of samples submitted as part of reproductive failure investigations. In Northern Italy, Bertasio et al. [21] tested >130,000 swine serum samples collected between 2002 and 2017, reporting an overall seroprevalence of 13%. Lee et al. [22] collected serum samples from 1959 swine prior to slaughter at various slaughterhouses in Vietnam, reporting an overall seroprevalence of 8.2%, lower than that reported previously by Boqvist et al. [23].

In Mainland China, implementation of various control and preventative measures have resulted in decreased leptospirosis incidence within the human population [24,25]. In the Pearl River Basin region (China), Dhewantara et al. [25] reported an association between leptospirosis incidence in people and livestock density, while leptospirosis was more common in farmers (type of farmer was not defined). Rodents are reported reservoirs for infection [25,26], with the results of Yalin et al. [26] suggesting they are the main reservoir of infection in the Jiangxi Province (China). Yalin et al. [26] also tested samples from 50 swine in the province for evidence of *Leptospira*, with only one sample testing positive.

Recently, leptospirosis infection of dogs has caused concern in the HKSAR [27]; however, the leptospiral status of other animals, including farmed swine, is unknown. At present, there are 40 active commercial swine farms operating in the HKSAR. Vaccination for leptospirosis is not undertaken by these swine farmers, while anecdotal reports suggest clinical leptospirosis is not a problem on-farm. The present cross-sectional study was undertaken to investigate the leptospirosis status of farmed swine in the HKSAR and to determine the need for further investigation and/or interventions.

## 2 Materials and methods

### 2.1 Swine samples

There are 40 commercial swine farms currently operating in the HKSAR, of which seven farms were convenience sampled from January to August 2020 and included in the present study. The farms ranged in size from 34 to 500 breeding sows (average 267 sows), with all included farms raising swine from farrowing to slaughter. The swine farms in the HKSAR are intensive facilities where the swine are kept indoors. However, farms rely on natural ventilation with areas of the barns open to the outdoors. This means there is potential for rodents to enter the barns and/or move between barns and/or areas outside of individual farms.

On each farm, ten clinically healthy multiparous sows and five clinically healthy 12-week-old weaners were blood sampled via jugular or cranial vena cava venepuncture. All samples for each farm were collected by the veterinary researchers on the same day, with swine selected from the swine present in each respective age-group. The swine were not individually identified, so to sample, the researchers selected swine from those in the respective age group until the required number of samples were collected.

In addition, in the period from January to October 2020, 64 swine of varying ages were submitted to the veterinary research team for post-mortem examination as part of a clinical service. These swine were either clinically ill and subsequently euthanised (*n* = 55), or had died on farm and were collected within two hours of death (*n* = 9). Swine were submitted from 26 farms (average 2.4 per farm; range 1–6). As part of the standard post-mortem protocol, a kidney sample was collected from each.

All animal samples collected in the present study were collected in compliance with the HKSAR guidelines for research on animals, with relevant animal licences and animal ethics approval; City University Animal Ethics Approval, A-0402 (Improving Pig Health and Production in Hong Kong).

### 2.2 Blood sample processing and microscopic agglutination test (MAT)

Each blood sample was collected into a 6 mL plain vacutainer (Vacuette©). Blood samples were transported to the laboratory (CityU Veterinary Diagnostic Laboratory (VDL), City University of Hong Kong) within two hours of collection. Serum was separated by centrifugation, after which 2 mL of serum was pipetted and stored in a Thermo Scientific Revco freezer at -30 °C, until all 105 swine samples were collected. Serum samples were then forwarded as a batch in a 19 L dry ice shipper filled with dry ice, via direct air door to door service (SFS Pharma), to the reference laboratory in Thailand.

The MAT was performed at the reference laboratory in Thailand, using the standard method described by the WHO/FAO/OIE [28], with a panel of 24 reference *Leptospira* serovars including *L interrogans* serogroups: Autumnalis, Ballum, Bataviae, Bratislava, Canicola, Celledoni, Cynopteri, Djasiman, Grippotyphosa, Hebdomadis, Icterohaemorrhagiae, Javanica, Louisiana, Manhao, Mini, Panama, Pomona, Pyrogenes, Ranarum, Sarmin, Sejroe, Shermani, Tarassovi and *L biflexa* serovar Patoc. Serum dilutions began at 1:25 and were tested and compared with control cultures diluted ½ in phosphate buffered saline. Serum samples with the minimum titre of 1:100 were considered positive.

### 2.3 Kidney sample processing and polymerase chain reaction (PCR)

Fresh kidney samples (*n* = 64) were tested for *Leptospira* using polymerase chain reaction (PCR) at VDL. DNA extraction and amplification were performed using the Qiagen QIAamp DSP Virus Kit (Qiagen, Hilden, Germany) and the outer membrane protein lipl32 genesig Advanced Kit following the manufacturer's protocol (Primerdesign, Chandler's Ford, United Kingdom). All sets of extracts included negative extraction controls of molecular-grade water. Samples were either tested immediately or stored at 4 °C prior to testing*.* The kit used detects 29 serovars (Supplementary Information) from the *Leptospira* genus.

### 2.4 Statistical analysis

Bayesian methods were used to compute the probability that the population prevalence of leptospirosis in farmed swine in the HKSAR was less than a specified value (3%), given our observed serological results for all sampled swine (X), namely, P(*p* < 0.03|X = x). In order to compute this probability, the prior understanding of the population prevalence was required. However, there was no existing reliable data available. Therefore, the best estimate of the prevalence was set at 3% consistent with the prevalence level in which we were interested; this was interpreted as the most likely value (the mode) before observing any data. To reflect our uncertainty about this estimate, we were 99% certain that the prevalence was lower than 10% (0.99-quantile); a conservative estimate, as a 10% prevalence was very unlikely in the HKSAR, particularly given the lack of clinical cases reported. This information was then converted to a beta distribution, i.e. Beta (3.765, 90.398) by a numerical method (technical details are presented in Appendix A).

The distribution of p given the observed data (the posterior distribution) was Beta (3.765, 195.398), derived based on the Bayes' theorem (details available in Appendix B). Based on this posterior distribution, the posterior prevalence estimate was firstly computed. Eventually the probability that the population prevalence was <3%, given the observed MAT results, was computed by integrating (numerically) the area under the PDF curve of the posterior distribution from 0 to 0.03.

Sensitivity analysis is required in every Bayesian analysis, meaning different, but not radically different, priors should be used in the analysis [29]. In this sensitivity analysis we kept the 0.99 quantile fixed and decreased and increased our best estimate by up to 25%, i.e. the lower and upper limits of our best estimate would be 0.024 and 0.036. Then a list of numbers within this range was created, starting at 0.024 and increasing by 0.002 increments. The corresponding probabilities of prevalence being <3% given the MAT results were then computed.

## 3 Results

### 3.1 Microscopic agglutination test (MAT) and polymerase chain reaction (PCR)

Of the 105 swine sera tested by MAT, no positive sera were detected at the 1:100 titre to any of the 24 serovars tested. In addition, *Leptospira* DNA was not detected in any of the 64 kidney samples.

### 3.2 Bayesian analysis

**Fig. 1:**
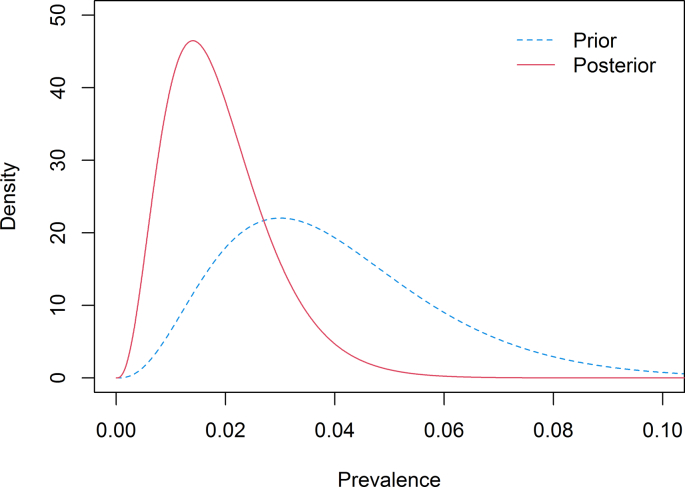
The prior and posterior distributions of prevalence of leptospirosis in farmed swine in the Hong Kong Special Administrative Region (HKSAR), where the posterior prevalence is determined using the observed data (no positive out of the 105 swine tested via Microscopic Agglutination Test (MAT)).

Under our main prior, the median posterior prevalence was 1.7% with 95% probability interval (PI): 0.5%–4.2%. This could be interpreted as the probability that the prevalence of leptospirosis in the farmed swine population in the HKSAR falls in the range 0.5%–4.2% was 95%. This posterior distribution was different from our prior distribution for the prevalence, suggesting new information was obtained by performing the data analysis (Fig. 1). Given the observed MAT serological results, the probability of the leptospirosis prevalence being <3% in the farmed swine population in the HKSAR was 87.4%. This could be interpreted as after observing zero positive out of 105 swine tested by MAT, we were 87.4% certain that the population prevalence of leptospirosis was less than 3% in the farmed swine population in the HKSAR.

The result of the sensitivity analysis is shown in Table 1. This result suggests that we became more and more certain (from 87.4% to 92.7%) to declare the leptospirosis prevalence <3% if our best prevalence estimate (pre-test) decreased from 3% to 2.4%; while we became less and less certain (from 87.4% to 78.2%) to make such declaration if our best estimate increased from 3% to 3.6%. The result of this sensitivity analysis could be explained, as increasing the most likely value of the prior prevalence while holding constant 0.99-quantile induced a more concentrated prior probability distribution, which would then have stronger impact on the posterior inferences. The posterior prevalence estimates indicate that given any of the selected prior distributions, it was more than 97.5% certain that the prevalence of leptospirosis in the farmed swine population in the HKSAR was no more than 5%.

**Table 1:** Sensitivity analysis for prevalence of leptospirosis in farmed swine in the Hong Kong Special Administrative Region (HKSAR), where best estimate (3%, 0.03) was decreased and increased by up to 25% (2.4%, 0.024 to 3.6%, 0.036).

Best estimate of the prevalence before observing the data	Prevalence estimated after observing the data	Probability that the prevalence of leptospirosis is < 3%
0.024	0.014, 95%PI: 0.003–0.038	92.7%
0.026	0.015, 95%PI: 0.004–0.039	91.2%
0.028	0.016, 95%PI: 0.004–0.04	89.5%
0.03	0.017, 95%PI: 0.005–0.042	87.4%
0.032	0.019, 95%PI: 0.006–0.043	84.8%
0.034	0.02, 95%PI: 0.007–0.045	81.8%
0.036	0.02, 95%PI: 0.008–0.047	78.2%

## 4 Discussion

The widely accepted minimum final dilution microscopic agglutination test (MAT) titre considered to be significant is 1:100, with titres ≥1:100 considered positive [11,[20], [21], [22]]. Using this titre as the cut-off, there was no serological evidence of active *Leptospira* infection or previous seroconversion in any of the 105 swine included in the present serological survey. Additionally, none of the 64 swine submitted for post-mortem had molecular evidence of infection in their kidneys', where leptospires persist in chronically infected animals [14]. Given that none of the 105 swine sampled were positive on MAT, we then decided to compute the probability that the prevalence of leptospirosis was <3%. We selected 3% as the threshold prevalence as we considered it unlikely that leptospirosis would be endemic in the HKSAR at a seroprevalence lower than this. This assumption was based on reported seroprevalences from swine overseas, including 8.2% to 73.0% in Vietnam [22,23,30], 11.3% in Thailand [31], 13.0% in Italy [21] and 20.2% in Germany [20]. This 3% threshold also allowed us a high degree of certainty in our interpretation and conclusions, rather than trying to declare “disease freedom” where the population prevalence is smaller than a very small (for example <0.1%) threshold prevalence [32]. Declaration of disease freedom would have required a greater number of swine samples than were available in the present study. For example, to achieve the same level of certainly as our present analysis, but instead using a prevalence threshold of <0.1%, we would have required 5900 swine samples.

Bayesian methods were chosen in this study to further investigate the probability of a negligible infection scenario, given our serological results. In this case, negligible means <3% prevalence of leptospirosis in farmed swine in the HKSAR. This method was selected over the traditional sampling for detection of disease method described by Fosgate [32], as a hypothesis testing based method may lead to difficulty in the interpretation of the result. In this study we were able to make a probabilistic statement about the prevalence of leptospirosis after observing our results (no positives) to provide a logical and coherent interpretation for declaring disease status that can be understood by farmers and industry professionals alike.

Using Bayesian methods was appealing, however, the fact that the parameter (i.e. the prevalence) was modeled as a random variable required a probability statement about it before we had observed the data. This probability statement is often referred to as the prior distribution, and represents beliefs about the likely values of the prevalence prior to consideration of the data. Selecting a sensible prior distribution is important to obtain a meaningful posterior inference. If a disinformative prior is selected, in this case using values that are unrealistic to the leptospirosis situation in farmed swine the HKSAR, the posterior inferences would be meaningless. One might propose the use of a uniform prior such as Beta (1, 1) in our analysis, as such a prior is diffuse and considered to be “non-informative”, which might be appropriate as no studies have ever been conducted to investigate the leptospirosis prevalence in farmed swine in the HKSAR [33]. However, using this uniform prior would mean we assume that the mean prevalence was 50%, and that it was equally likely that all or none of the swine were infected. One would therefore highly doubt the validity of using such prior for our analysis. Hence, after consideration, we preferred to use our informative priors. As one can also see in the sensitivity analysis, we only increased/decreased the prior mode by up to 25% (as per Johnson et al. [29] perturbing the prior mode by modest amounts) rather than modifying the 0.99-quantile. Increasing the 0.99-quantile from 10% to a bigger value was considered unrealistic for the HKSAR situation; while decreasing the 0.99-quantile resulted in a more concentrated prior, which was not recommended by Johnson et al. [29]. Certainly, we acknowledge that increasing the prior mode also produced a more concentrated prior, which led to a more conservative posterior probability. This was not harmful; instead, it led to interpretation of the results in a more cautious manner.

The present study included blood samples from 105 swine from two different age groups from each of seven farms. The seven farms were selected based on convenience, selecting farms willing to participate in the study. Although this may have biased the sampling, there were no clinical reports or prior information on the leptospirosis status of any farms and no farms were known to deal with leptospirosis disease in their swine for any farms in the HKSAR. Additionally, although this is a limited number of samples and farms, the inclusion of seven farms in the serological survey represents 17.5% of farms (7/40) in the HKSAR. When using the MAT, it is recommended that at least 10 swine, or 10% of swine (whichever is greater) are included [12]. In this study, these guidelines were met for 3/7 farms when considering the sow herd, however, economic and operational constraints meant we were unable to sample more swine in the larger herds. Seroconversion is more likely in older swine [23,34], so the inclusion of adult sows in the present study made it more likely seroconversion would be observed. The authors acknowledge that this may have biased the results, however, it also means we have greater confidence in our interpretation as no positives were observed in these sows. Despite sampling multiple different farms, the variability of the within-farm prevalence was not considered, as the observed serological results (zero positives) provided no information regarding the distribution of these prevalences [35]. Therefore, in the present study all samples were considered together, regardless of farm and age group.

To conclusively answer the question of prevalence of leptospirosis in farmed swine in the HKSAR, further sampling of a larger number of swine from all farms in the HKSAR would be needed. However, the lack of reports of clinical leptospirosis in swine in the HKSAR, combined with the results from the present study, mean this is not a priority. At present, leptospirosis in farmed swine in the HKSAR appears to be of no significance. However, it is important to note the possible consequences of introduction of *Leptospira* to naïve populations, namely, reduced reproductive performance and clinical cases. It is also prudent for those working within the swine producing industry to maintain an awareness of leptospirosis due to the potential for zoonotic disease.

## 5 Conclusions

There was no evidence of *Leptospira* infection in the swine in this study. Based on this and the computed probabilities, it is highly likely that the prevalence of leptospirosis in farmed swine in the HKSAR is negligible. Therefore, at this point in time leptospirosis is not a priority for swine veterinarians and commercial farmers in the HKSAR. However, it would be prudent for those working within the swine industry to maintain ongoing awareness due to the potential for zoonotic disease combined with possible consequences of introduction to naïve populations.

## Funding

This work was supported by the Sustainable Agricultural Development Fund under the Agriculture, Fisheries and Conservation Department of the Government of the HKSAR, SADF 0009 – “Improving Pig Health and Production in Hong Kong”.

## Declaration of Competing Interest

The authors declare no conflict of interest.

## Figures and Tables

**Figure:**
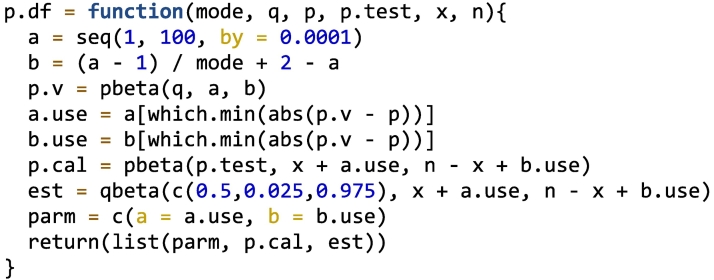


## Appendix A Find the parameters for a beta random variable based on the prior knowledge

A beta random variable Y has two parameters a and b. To find the best beta random variable to describe our prior knowledge, we followed these steps:

First, a list of numbers starting from 1 to 100 with step size =0.0001 was created.
Second, at the given mode, the corresponding b was then calculated.
Third, at the given 0.99-quantile (q_0.99_), a list of P(Y < q_0.99_) were computed.
Fourth, the absolute value of the difference between the P(Y < q_0.99_)s and 0.99, i.e. |P(Y < q_0.99_) - 0.99|, were computed.
Fifth, the minimum value of these absolute differences was identified.
Sixth, the beta random variable with the min(|P(Y < q_0.99_) - 0.99|) was selected as the best approximation of our prior knowledge.

The R code is provided:

## Appendix B Deriving the posterior distribution

The number of MAT positive swine out of n swine was modeled as *X*~Bin (*n*, *p*); and the prevalence of leptospirosis was modeled as *p*~Beta (*a*, *b*).

The probability density function for a beta random variable is:

$$f\left(p\right)=\left\{\begin{array}{c} \frac{\Gamma \left(a+b\right)}{\Gamma \left(a\right)\Gamma \left(b\right)}p^{a-1}{\left(1-p\right)}^{b-1},0<p<1,\\ 0,\text{otherwise}. \end{array}\right)$$

$$∵\int_{0}^{1}\frac{\Gamma \left(a+b\right)}{\Gamma \left(a\right)\Gamma \left(b\right)}p^{a-1}{\left(1-p\right)}^{b-1}\mathrm{dp}=1$$

$$∴\frac{\Gamma \left(a\right)\Gamma \left(b\right)}{\Gamma \left(a+b\right)}=\int_{0}^{1}p^{a-1}{\left(1-p\right)}^{b-1}\mathrm{dp}$$

The probability mass function for a binomial random variable is:

fxp=nxpx1−pn−x,x=0,1,2,…,n

Based on the Bayes' theorem, the posterior probability distribution of *p* is:

fpx=fxpfp∫01fxpfpdp=Γa+bΓaΓbnxpx+a−11−pn−x+b−1∫01Γa+bΓaΓbnxpx+a−11−pn−x+b−1dp=Γa+bΓaΓbnxpx+a−11−pn−x+b−1Γa+bΓaΓbnx∫01px+a−11−pn−x+b−1dp=px+a−11−pn−x+b−1∫01px+a−11−pn−x+b−1dp

$$=\frac{p^{c-1}{\left(1-p\right)}^{d-1}}{\int_{0}^{1}p^{c-1}{\left(1-p\right)}^{d-1}\mathrm{dp}}\;\left(\mathrm{Let}\;c=x+a\;\text{and}\;d=n-x+b\right)$$

$$=\frac{p^{c-1}{\left(1-p\right)}^{d-1}}{\frac{\Gamma \left(c\right)\Gamma \left(d\right)}{\Gamma \left(c+d\right)}}=\frac{\Gamma \left(c+d\right)}{\Gamma \left(c\right)\Gamma \left(d\right)}p^{c-1}{\left(1-p\right)}^{d-1}$$

$$=\frac{\Gamma \left(n+a+b\right)}{\Gamma \left(x+a\right)\Gamma \left(n-x+b\right)}p^{x+a-1}{\left(1-p\right)}^{n-x+b-1}$$

It can be seen that the posterior distribution of *p* is also a beta random variable with parameter *x* + *a* and *n* − *x* + *b*.

## Appendix C Supplementary data

Supplementary material Image 1
