# Supplementary material for: Absence of serological or molecular evidence of Leptospira infection in farmed swine in the Hong Kong Special Administrative Region
Source: One Health. 2021 Aug 30;13:100321. doi: 10.1016/j.onehlt.2021.100321 (PMC8411228; doi:10.1016/j.onehlt.2021.100321)
Supplement: Supplementary file 1 — Supplementary material [file mmc1.docx]

**Supplementary information**

Leptospira organisms detected by Genesig PCR

*Leptospira borgpetersenii* serovar Ballum

*Leptospira borgpetersenii* serovar Castellonis

*Leptospira borgpetersenii* serovar Javanica

*Leptospira interrogans* serovar Copenhageni

*Leptospira interrogans* serovar Copenhageni/Icterohaemorrhagiae

*Leptospira interrogans* serovar Hardjo-prajitno

*Leptospira interrogans* serovar Manilae

*Leptospira interrogans* serovar Linhai

*Leptospira interrogans* serovar Autumnalis

*Leptospira interrogans* serovar Balico

*Leptospira interrogans* serovar Lai

*Leptospira interrogans* serovar Icterohaemorrhagiae

*Leptospira interrogans* serovar Wolffi

*Leptospira interrogans* serovar Paidjan

*Leptospira interrogans* serovar hardjo

*Leptospira interrogans* serovar Sejroe

*Leptospira interrogans* serovar lai

*Leptospira interrogans* serovar Pomona

*Leptospira interrogans* serovar Bratislava

*Leptospira interrogans* serovar Canicola

*Leptospira interrogans* serovar Jalna

*Leptospira interrogans* serovar Grippotyphosa

*Leptospira interrogans* serovar Hebdomadis

*Leptospira interrogans* serovar Mini

*Leptospira interrogans* serovar Grippotyphosa

*Leptospira interrogans* serovar Pyrogenes

*Leptospira kirschneri* serovar Tsaratsovo

*Leptospira kirschneri* serovar Mozdok

*Leptospira kirschneri* serovar Altodouro

*Leptospira santarosai* serovar Shermani

*Leptospira weilii* serovar Manhao
